# Exposure to Endocrine Disrupters and Nuclear Receptor Gene Expression in Infertile and Fertile Women from Different Italian Areas

**DOI:** 10.3390/ijerph111010146

**Published:** 2014-09-29

**Authors:** Cinzia La Rocca, Sabrina Tait, Cristiana Guerranti, Luca Busani, Francesca Ciardo, Bruno Bergamasco, Laura Stecca, Guido Perra, Francesca Romana Mancini, Roberto Marci, Giulia Bordi, Donatella Caserta, Silvano Focardi, Massimo Moscarini, Alberto Mantovani

**Affiliations:** 1 Department of Food Safety and Veterinary Public Health, Istituto Superiore di Sanità, Roma 00161, Italy; E-Mails: sabrina.tait@iss.it (S.T.); luca.busani@iss.it (L.B.); brn_001@hotmail.it (B.B.); laurastecca@hotmail.it (L.S.); francesca.r.mancini@gmail.com (F.R.M.); alberto.mantovani@iss.it (A.M.); 2 Department of Physical, Earth and Environmental Sciences, University of Siena, Siena 53100, Italy; E-Mails: guerranticri@unisi.it (C.G.); guido.perra@alice.it (G.P.); focardi@unisi.it (S.F.); 3 Institute of Clinical Physiology, National Research Council, Pisa 73100, Italy; 4 Department of Obstetric and Gynecological Sciences and Urological Sciences, University of Roma Sapienza, S. Andrea Hospital, Roma 00189, Italy; E-Mails: ciardofrancesca@libero.it (F.C.); bordi.giulia@gmail.com (G.B.); donatella.caserta@uniroma1.it (D.C.); massimo.moscarini@uniroma1.it (M.M.); 5 Department of Veterinary Medicine, University of Bologna, Ozzano dell’Emilia 40064, Italy; 6 Department of Biomedical Sciences and Advanced Therapies, Section of Obstetrics and Gynaecology, University of Ferrara, Ferrara 44121, Italy; E-Mail: roberto.marci@unife.it

**Keywords:** human exposure, women infertility, PFOS, PFOA, BPA, DEHP, MEHP, biomarkers

## Abstract

Within the PREVIENI project, infertile and fertile women were enrolled from metropolitan, urban and rural Italian areas. Blood/serum levels of several endocrine disrupters (EDs) (perfluorooctane sulfonate, PFOS; perfluorooctanoic acid, PFOA; di-2-ethylhexyl-phthalate, DEHP; mono-(2-ethylhexyl)-phthalate, MEHP; bisphenol A, BPA) were evaluated concurrently with nuclear receptors (NRs) gene expression levels (ERα, ERβ, AR, AhR, PPARγ, PXR) in peripheral blood mononuclear cells (PBMCs). Infertile women from the metropolitan area displayed significantly higher levels of: BPA compared to fertile women (14.9 *vs.* 0.5 ng/mL serum); BPA and MEHP compared to infertile women from urban and rural areas; enhanced expression levels of NRs, except PPARγ. Infertile women from urban and rural areas had PFOA levels significantly higher than those from metropolitan areas. Our study indicates the relevance of the living environment when investigating the exposure to EDs and the modulation of the NR panel in PBMC as a suitable biomarker of the effect, to assess the EDs impact on reproductive health.

## 1. Introduction

Infertility is defined as “the failure to achieve a clinical pregnancy after 12 months or more of regular unprotected sexual intercourse” [1]. This condition affects millions of women of reproductive age worldwide; the prevalence depends on the residing geographic area, pointing to the role of environmental factors [2]. Several toxicological studies identified associations between exposure to endocrine disrupters (EDs) and women’s reproductive problems leading to infertility [3,4]. In addition, an increasing number of human biomonitoring (HBM) studies shows that the general population is exposed to persistent and bioaccumulating EDs [5,6]. On the contrary, limited HBM data still exist on several EDs that are widely present in foods, the living environment and consumer products, such as perfluorooctane sulfonate (PFOS), perfluorooctanoic acid (PFOA), di-2-ethylhexyl phthalate (DEHP), as well as bisphenol A (BPA).

PFOS and PFOA are still widely used in paper, food packaging contact materials and textiles [7], and since 2010, they have been included among persistent organic pollutants (POPs) [8]. Human exposure occurs mainly through the diet, especially fish, but neither compound is routinely monitored in foods in Europe; indoor dust is also important for PFOA exposure [9,10]. PFOS and PFOA exposures have been associated with reduced fertility [11], longer time to pregnancy [12], as well as endometriosis [13]. Both chemicals enhance the activity of different nuclear receptors (NRs), including the estrogen receptor (ER) [14], the peroxisome proliferator-activated receptors (PPARs) [15,16] and the pregnane X receptor (PXR) [17].

DEHP is a common plasticizer used primarily in soft polyvinyl chloride; its presence in several consumer products (building materials, floorings, clothing, furnishings, food contact materials) leads to a widespread human exposure, even though DEHP is not considered a persistent compound [18]. Due to toxicological evidence, DEHP use has been recently restricted [19]. Upon intake, DEHP is quickly metabolized to its major toxic metabolite, mono-(2-ethylhexyl) phthalate (MEHP), representing the toxicologically relevant biomarker of DEHP exposure. DEHP is an agonist of PPARs and PXR, also altering the biosynthesis of estrogens and androgens. Epidemiological studies suggest a possible association with endometriosis, albeit the results are not univocal [20,21,22], whereas toxicological studies on rodents exposed to DEHP/MEHP point out impaired female reproductive function with decreased aromatase and estradiol levels [23]. 

BPA is extensively used as a monomer in polycarbonate plastics and in epoxy resins, representing one of the world’s highest production volume chemicals. A recent, comprehensive assessment by the European Food Safety Authority, currently available as draft, identifies food contact items, followed by thermal paper, as the main determinants of human exposure [24,25]. The adverse health effects of BPA are still a matter of intense debate. In women, BPA internal levels have been positively correlated with infertility-related conditions [26,27,28]. BPA is considered mainly as an ERα and ERβ agonist, but it can also affect other endocrine pathways, e.g., by acting as an antagonist of the androgen receptor (AR) or as an agonist of the aryl hydrocarbon receptor (AhR), involved in cross-talk processes with ERs, AR and other NRs [29] and agonist of PXR [30]. 

PFOS, PFOA, DEHP and BPA are the target EDs of the project, PREVIENI (Study in model areas on the environmental and health impact of some emerging chemical contaminants (endocrine disrupters): living environment, reproductive outcomes and repercussions in childhood; http://www.iss.it/prvn, supported by the Italian Ministry of Environment). The project’s first results pivoted on the possible relationship between EDs and reproductive health status. The PREVIENI cohort of Italian infertile women had a higher presence of detectable BPA serum levels, as well as enhanced expression of ERα, ERβ, AR and PXR in peripheral blood mononuclear cells (PBMCs) in comparison with fertile controls: significant correlations were also observed between ERα, ERβ, AR, AhR and PXR expression levels and BPA, MEHP concentrations and between AhR and PFOA [31]. Within PREVIENI, fertile and infertile women were enrolled in three Italian areas representing different living environment scenarios, which may be related to different EDs exposure patterns: Roma (Lazio, Central Italy), with all of the features of a metropolitan environment and lifestyle; Ferrara (Emilia-Romagna, Northern Italy), a medium-sized town amid a prosperous area with many farms and small- or medium-sized industries; Sora (Lazio, Central Italy), a rural municipality characterized by intensive agricultural activities. Therefore, the goal of the present study is to assess whether the area of residence can be related to any difference in serum ED concentrations and gene expression levels of NRs in women with different reproductive health status.

## 2. Methods

### 2.1. Areas under Study

Three different geographic areas were considered in this study: a metropolitan area (Rome, Lazio Region, Central Italy, approximately 2,700,000 residents); a medium-sized urban area (Ferrara, Emilia-Romagna Region, Northern Italy, approximately 130,000 residents); and a rural area (Sora, Lazio Region, Central Italy, approximately 26,000 residents). In order to characterize the areas under study, territorial, demographic and productive indicators were chosen for their potential contribution to the environmental contamination as regards the EDs considered in this study. Data on the selected indicators for each area were obtained for the year 2011 from the Italian National Institute of Statistics (ISTAT, http://www.istat.it). 

### 2.2. Study Subjects

From January 2009, to December 2011, on a voluntary basis, 110 infertile and 43 fertile women were enrolled in the following medical centers per area:

- n = 49 infertile and n = 13 fertile women in the Department of Women Health and Territorial Medicine of “Sapienza” University “Sant’Andrea” Hospital, Rome;
- n = 38 infertile and n = 22 fertile women in the Department of Biomedical Sciences and Advanced Therapies, Section of Obstetrics and Gynaecology, University of Ferrara;
- n = 23 infertile and n = 8 fertile women in the Infertility Center S.T.S. (Sterility Therapy and Study) of Sora.

The fertile women were selected among those with a regular menstrual cycle who obtained a spontaneous pregnancy in the last year and stopped breastfeeding at least six months before starting the study. The infertile women, selected among those with a diagnosis of primary infertility (tubal infertility, endometriosis, anovulation, immunological factors) or unexplained infertility, were enrolled in the study before starting the infertility treatment. Inclusion criteria were: residing in the municipalities included in the area, age from 18 to 40 years, body mass index (BMI) <30 and PBMCs levels within the range of normal values for age and sex. 

The exclusion criteria, which included the main confounders, were: occupational exposure to the selected EDs (plastic, housewares or textile industries), smoking habit, vegetarian diet, BMI >30 and the evidence of inflammatory or infectious diseases. The study has been carried out in accordance with The Code of Ethics of the World Medical Association (Declaration of Helsinki). Approval from the Ethical Committees of the responsible structures of the IVF centers were obtained before the beginning of this study, and all enrolled women gave informed consent to study inclusion.

### 2.3. Collection and Storage of Samples 

All samples obtained from infertile women were collected before hormonal stimulation. Glass vials were used in order to avoid the possible release of DEHP or BPA from plastics. Three aliquots of venous blood were collected from each woman. For ED level determination, 5 mL of heparin-treated whole blood and 10 mL centrifuged blood to obtain serum were sampled and sent to the Environment Science Department “G. Sarfatti” (now the Department of Physical, Earth and Environmental Sciences) of the University of Siena.

For NR gene expression, blood samples were collected in PAXgene Blood RNA Tubes (PreAnalitiX, Plymouth, U.K.) and frozen until use. All samples were sent to the Food and Veterinary Toxicology Unit (Istituto Superiore di Sanità, Roma). Twelve samples from infertile subjects (eight from Roma, three from Ferrara and one from Sora) and two samples of fertile women from Ferrara were not analyzed for NR gene expression, due to delivery problems.

### 2.4. Chemical Analysis of Biomarker of Exposure 

Based on established literature methods (see below), BPA, DEHP and MEHP were measured in serum and PFOS and PFOA in whole blood. All analyzed EDs were extracted using a liquid-liquid separation procedure and measured using high performance liquid chromatography with electrospray ionization tandem mass spectrometry (LC-ESI-MS). 

#### 2.4.1. PFOS/PFOA

For analysis of PFOS/PFOA, an extraction was performed according to the analytical procedure previously described [32]. Briefly, the samples were extracted with methyl tert-butyl ether (MTBE, J.T. Baker). The solvent was evaporated under nitrogen and replaced with methanol (J.T. Baker). Twenty µL were injected into HPLC (equipped with Betasil© C18 column, Thermo Electron Corporation) interfaced to a mass spectrometer at linear triple quadrupoles, by an electrospray ionization (ESI) source, working in negative ion mode (Finnigan LTQ Thermo Electron Corporation, San Jose, CA). The limit of detection (LOD) for both PFOS and PFOA was 0.4 ng/mL, corresponding to the value of the compounds in the blanks +3 SD.

#### 2.4.2. DEHP/MEHP

The analytical procedure for the extraction of DEHP and MEHP from serum samples has been previously described [31]. Briefly, 0.5 g of each thawed sample were added to 4 mL of acetone (J.T. Baker), sonicated for 2 min and centrifuged for 15 min at 3000 rpm for two times. Supernatants were evaporated in a centrifugal evaporator (Thermo Scientific) and suspended with 0.5 mL of deionized water and 4 mL of acetic acid (J.T. Baker). After adjusting the volume to 0.5 mL, 5 μL of the sample were injected into the LC-ESI-MS system. A reverse phase HPLC column (Wakosil3C18, 2.0 × 100 mm, 3 μm; Wako Pure Chemical Industries Ltd.) was used. ESI-MS was operated in negative or positive ion mode depending on the analyte. The LODs were 2 ng/mL for MEHP and 10 ng/mL for DEHP. 

#### 2.4.3. BPA

Total BPA (free plus glucuronated) in serum was analyzed according to the procedure previously described [31]. Each aliquot of 0.5 mL of serum was incubated with 2 μL/mL of the enzyme I glucuronidase (Sigma-Aldrich) at 37 °C for 12 h. Subsequently, the sample was added to 3 mL of ethyl ether (J.T. Baker), shaken for 30 minutes and centrifuged at 4,000 rpm for 5 minutes. The procedure was repeated three times. The collected supernatants were then evaporated and reconstituted in 0.5 mL of methanol. Twenty microliter of sample were injected into a Betasil C18 column 50 × 2.1 mm at a flow rate of 250 μL/min in the HPLC-ESI-MS instrument. The negative ion for the identification of BPA was obtained by fragmentation of the ion 227 with collision energy of 35 and production of the ion (m/z) 212. The ESI source was set at a voltage of 5 kV and to a rush of 3 μA. The LOD was 0.5 ng/mL.

#### 2.4.4. Data Quality Assurance and Quality Control

The analytical protocol comprised measures to avoid contamination from plasticizers in test materials, which included, besides the use of metal needles and glassware vials for collection and storage of samples, the use of glass labware rinsed by acetone and hexane to remove potential contaminants and the assessment of method blanks [33].

In addition, in order to monitor and evaluate any possible contamination of samples, data quality assurance and quality control protocols were performed, including matrix spikes, laboratory blanks and continuing calibration verification. In particular, blanks were analyzed with each set of five samples to check possible iatrogenic contamination and interferences: levels of chemicals in such samples resulted in being below the limit of detection for each compound.

### 2.5. Gene Expression Analysis of Nuclear Receptors

Blood samples collected in PAXgene Tubes were extracted for their RNA content by the PAXgene Blood RNA Kit (Qiagen). Total RNA was quantified by NanoDrop (Thermo Scientific Wilmington, DE, USA) and assessed for its quality by 1% agarose gel electrophoresis; all of the samples were optimal to be further analyzed. For each sample, 1 μg of RNA was reverse transcribed to cDNA by the cDNA Synthesis Kit (Quantace, London, UK), according to the manufacturer’s protocol.

Gene expression analysis was performed by quantitative real-time PCR using the Sensi Mix SYBR Kit (Quantace), with GAPDH as the reference gene. Specific primers for the selected NRs and GAPDH were designed using the Primer-BLAST web application (http://www.ncbi.nlm.nih.gov/tools/primer-blast) and are listed in Table 1.

**Table 1:** Primers sequences, accession numbers and amplicon lengths for reference (GAPDH) and nuclear receptors (NR) genes.

Gene	RefSeq Accession		Sequence 5’ to 3’	Amplicon Length (bp)
GAPDH	NM_002046.4	forward	ACTCCTCCACCTTTGACGCT	273
		reverse	CTTCAAGGGGTCTACATGGC	
ERα	NM_000125.3	forward	ACTGCGGGCTCTACTTCATC	275
		reverse	GGCTGTTCCCAACAGAAGAC	
ERβ	NM_001040275.1	forward	CTCTTTTGCCTGAAGCAACG	269
		reverse	CTGGGCAGTTAAGGAGACCA	
AR	NM_000044.3	forward	CCCATCTATTTCCACACCCA	259
		reverse	GCAAAGTCTGAAGGTGCCAT	
PPARγ	NM_138712.3	forward	GATGACAGCGACTTGGCAAT	269
		reverse	AGGAGCGGGTGAAGACTCAT	
AhR	NM_001621.4	forward	TTCCACCTCAGTTGGCTTTG	233
		reverse	GGACTCGGCACAATAAAGCA	
PXR	NM_003889.3	forward	GGCCACTGGCTATCACTTCA	343
		reverse	GGTTTTCATCTGAGCCTCCA	

Amplification efficiencies, cDNA input dilution and primer concentrations were optimized by the standard curve method. Real-time PCR reactions were run on a Stratagene MP3005P Thermocycler. Experiments were performed in duplicate on 96-well PCR plates. The thermal program was as follows: 1 cycle at 94 for 10 min; 40 cycles at 94 °C for 10 s, 58 °C for 10 s and 72 °C for 10 s; and 1 dissociation cycle from 55 to 94 °C to verify amplification products. Data are expressed as 2^-ΔCt^ (ΔCt = Ct_TG_ – Ct_RG_), with Ct_TG_ as the threshold cycle of the target gene and Ct_RG_ as the threshold cycle of the reference gene.

### 2.6. Statistical Analysis

We performed statistical descriptive and comparative analysis using non-parametric tests. We decided to limit the statistical inference to single variable analyses (univariate statistics) stratifying by areas to provide unbiased results, taking into account the number of samples.

The concentration of each ED below the respective LOD has been considered as “<LOD”. For the inferential analyses and comparisons, the values below LOD have been replaced by half the LOD value (medium-bound) [34].

Dichotomous variables for concentrations of the EDs (0 if ≤ LOD and 1 if > LOD) were created. ED concentrations and NR expression values were not normally distributed, and the log transformation did not normalize the distributions. Therefore, differences between infertile and fertile women resident in the same area and across the areas were assessed with the Wilcoxon–Mann–Whitney test, adjusting for multiple comparisons using the Bonferroni procedure for correcting the *p*-value. 

The risk of infertility in relation to the ED concentration was calculated stratifying by area of residence using univariate analysis and the chi-square test.

Statistical analysis was performed with STATA 11.2 (StataCorp, 4905 Lakeway Drive, College 17 Station, TX, USA) setting significance at *p* < 0.05.

**Table 2:** Distribution of a set of territorial, demographic and productive indicators in the study areas. Data from the Italian National Institute of Statistics (ISTAT).

Areas	Metropolitan (Rome)		Urban (Ferrara)		Rural (Sora)	
Indicators	1–10 employees	>10 employees	1–10 employees	>10 employees	1–10 employees	>10 employees
Agricultural enterprises	393	17	1684	10	4	0
Textile industries	206	9	40	2	4	0
Petroleum refinery	16	12	0	1	0	0
Manufactures of chemicals	121	43	12	8	4	0
Manufactures of articles of rubber	0	0	0	0	0	0
Manufacture of articles of plastics	0	0	0	0	0	0
Sanitation and waste management	39	6	2	0	0	0
Population	2,724,347		134,464		26,542	
Surface (km^2^)	1307.71		404.36		71.82	
Population density (inhabitants/km^2^)	2083.30		332.54		369.56	

## 3. Results

### 3.1. Areas Characterization

Considering the territorial, demographic and productive indicators, differences were evidenced in the three areas in the number and percentage of industries by category of production per km^2^. In particular, the metropolitan area was characterized by a high population density with about three million people and by the presence of agricultural and industrial enterprises. However, considering the population density, the highest proportion of enterprises with more than 10 employees was observed in the urban area. In the rural area, neither factories nor farms with more than 10 employees were reported (Table 2).

### 3.2. Biomarkers of Exposure

PFOS, PFOA, MEHP and BPA blood/serum levels in the women enrolled are summarized in Table 3. The results expressed as mean, median and interquartile range (25th–75th percentile) values are provided for both fertile and infertile groups by area. Since DEHP was found above the LOD only in one infertile woman in the metropolitan area (72.25 ng/mL) and in three infertile women in the rural area (range 10.03–25.33 ng/mL), it was excluded from the analysis.

**Table 3:** Analytical values of PFOS, PFOA (ng/mL blood), MEHP and total BPA (ng/mL serum) in enrolled women grouped by area of residence and subject group.

Chemicals		PFOS		PFOA		MEHP		BPA	
Areas		*infertile*	*fertile*	*infertile*	*fertile*	*infertile*	*fertile*	*infertile*	*fertile*
**Total**	mean	3.5	2.2	1.8	1.7	37.9	13.1	10.6	4.8
(110 infertile;	median	<0.4	<0.4	<0.4	<0.4	8.3	3.3	<0.5	<0.5
43 fertile)	25th p ^#^	<0.4	<0.4	<0.4	<0.4	<2	<2	<0.5	<0.5
	75th p	0.86	<0.4	3.7	3.3	26.8	11.3	9.4	<0.5
	%>LOD	30.00%	20.90%	40.90%	34.90%	62.70%	58.10%	41.80%	23.30%
**Metropolitan**	mean	6.9	4.5	0.6	<0.4	75.3	32.3	19.5	7.3
(49 infertile;	median	<0.4	<0.4	<0.4 ^a^	<0.4 ^a^	23.1 ^a^	12.1 ^a^	14.9 ^a,^*	<0.5 *
13 fertile)	25th p	<0.4	<0.4	<0.4	<0.4	<2	<2	<0.5	<0.5
	75th p	2.9	<0.4	<0.4	<0.4	127.4	18.2	25.8	<0.5
	%>LOD	30.6%	7.7%	10.2%	0.0%	69.4%	69.2%	71.4%	23.1%
**Urban**	mean	0.8	1.3	3.2	2.6	8.4	6.2	1.7	2.2
(38 infertile;	median	<0.4	<0.4	3.6 ^b^	1.1 ^b^	4.4 ^b^	3.7 ^a^	<0.5 ^b^	<0.5
22 fertile)	25th p	<0.4	<0.4	<0.4	<0.4	<2	<2	<0.5	<0.5
	75th p	0.6	<0.4	4.9	5.2	9.4	5.6	3.8	5.4
	%>LOD	26.3%	22.7%	71.1%	50.0%	73.7%	72.7%	26.3%	27.3%
**Rural**	mean	1	1.2	2.1	1.9	7.2	<2	6	7.8
(23 infertile;	median	<0.4	<0.4	2.2 ^b^	1.6 ^b^	<2 ^b^	<2 ^b^	<0.5 ^b^	<0.5
8 fertile)	25th p	<0.4	<0.4	<0.4	<0.4	<2	<2	<0.5	<0.5
	75th p	0.9	1.6	3.7	3.2	18.6	<2	<0.5	<0.5
	%>LOD	34.8%	37.5%	56.5%	50.0%	30.4%	0.0%	4.4%	12.5%

LOD = 0.4 ng/mL for PFOS and PFOA; 2 ng/mL for MEHP; 0.5 ng/mL for BPA. * indicates statistically significant different values between fertile and infertile women in the same area of residence (Mann-Whitney test corrected with the Bonferroni procedure). ^a,b^ Different superscript letters indicate statistically significant different values between areas within subjects of the same group (Mann-Whitney test corrected with the Bonferroni procedure). ^#^ 25th and 75th p indicate percentile values.

The percentage of subjects exposed to each specific ED (levels > LOD), as well as the corresponding concentrations were different in the three study areas. BPA was significantly more prevalent in the metropolitan area, with a significantly higher level in infertile women. MEHP was detected in over 65% of the women from both the metropolitan and urban areas, but levels were significantly higher in women residing in the metropolitan area; in the rural area, MEHP was found in about 22% of women, with significantly lower levels compared to the other areas. BPA was detected in over 60%, 25% and 6% of the women from metropolitan, urban and rural areas, respectively. PFOS was detected in about 30% of the subjects in each area without differences in concentration. PFOA was the only ED significantly more prevalent in the urban and rural areas: it was detected in over 50% of the women from the urban and rural areas, but in less than 10% of those from the metropolitan area, where levels were significantly lower than in the other two areas.

The comparison of ED levels between infertile and fertile women for each area revealed that in the metropolitan area, infertile women had significantly higher BPA levels than fertile women (median values 14.9 *vs*. 0.5 ng/mL serum).

The comparisons of ED concentrations between fertile women by area of residence showed no significant difference for BPA or PFOS concentrations. MEHP concentration was significantly lower in the rural area, while no difference was found between the urban and the metropolitan area. Regarding PFOA, fertile women in the urban and rural areas had significantly higher levels than those in the metropolitan area.

Similar to fertile women, the comparison between infertile women by area of residence showed no significant difference for PFOS internal levels. On the other hand, in the metropolitan area, significantly higher concentrations of MEHP and BPA and significantly lower concentrations of PFOA were found compared to the urban and rural areas, while no significant differences were highlighted between the rural and urban areas. By comparing the proportion of infertile and fertile women with EDs concentration >LOD in univariate analysis, a significant association with infertility was observed for BPA in the metropolitan area (OR = 8.3; 95% CI = 1.7–52.1) (Table 4).

**Table 4:** Infertility risk factors associated with endocrine disrupters (ED) exposure (serum concentration >LOD) in enrolled women grouped by area of residence.

Chemicals	Total (n = 153)			Metropolitan area (n = 62)			Urban area (n = 60)			Rural area (n = 31)		
	OR	95% CI		OR	95% CI		OR	95% CI		OR	95% CI	
PFOS	1.6	0.7	4.3	5.3	0.7	241.1	1.2	0.3	5.3	0.9	0.1	7.3
PFOA	1.3	0.6	2.9	ND	-	-	2.5	0.7	8.4	1.3	0.2	8.9
MEHP	1.2	0.6	2.6	1.0	0.2	4.4	1.1	0.3	3.9	ND	-	-
BPA	2.4 *	1.0	5.9	8.3 *	1.7	52.1	1.0	0.3	3.8	0.3	0.0	28.5

* Indicates a statistically significant value; ND indicates not determinable.

### 3.3. Nuclear Receptors Gene Expression

NRs gene expression values (mean, median and interquartile range) in infertile and fertile women by area are summarized in Table 5. 

**Table 5:** Gene expression values of NRs in enrolled women grouped by area of residence and subject group. Data are expressed as 2^ΔCt^ values with GAPDH as the reference gene.

Nuclear Receptors		ERα		ERβ		AR		PPARγ		AhR		PXR	
Areas		*infertile*	*fertile*	*infertile*	*fertile*	*infertile*	*fertile*	*infertile*	*fertile*	*infertile*	*fertile*	*infertile*	*fertile*
**Total**	mean	0.1138	0.0151	0.0937	0.0122	0.1086	0.0142	0.0003	0.0008	0.0256	0.0047	0.1015	0.0122
(98 infertile ;	median	0.0082	0.0007	0.0081	0.0017	0.0087	0.001	0.0001	0.0002	0.0021	0.0012	0.0067	0.0004
41 fertile)	25th p ^#^	0.0005	0.0003	0.0011	0.0009	0.0008	0.0005	0.0001	0.0001	0.0009	0.0007	0.0002	0.0002
	75th p	0.0636	0.0058	0.0413	0.0071	0.0636	0.0099	0.0003	0.0002	0.0081	0.0022	0.0998	0.0043
**Metropolitan**	mean	0.2582	0.0282	0.2143	0.0216	0.2439	0.0232	0.0004	0.0003	0.0589	0.0068	0.2231	0.0195
(41 infertile ;	median	0.0647 ^a,^*	0.0007 ^a,^*	0.0591 ^a,^*	0.0013 *	0.0593 ^a,^*	0.0008 *	0.0002	0.0002	0.009 ^a,^*	0.0013 *	0.0998 ^a,^*	0.0004 ^a,^*
13 fertile)	25th p	0.0256	0.0004	0.0132	0.0008	0.0186	0.0005	0.0000	0.0001	0.0041	0.0007	0.0170	0.0003
	75th p	0.2963	0.0059	0.2398	0.0098	0.2707	0.0099	0.0003	0.0002	0.0265	0.0039	0.2698	0.0043
**Urban**	mean	0.0158	0.0125	0.0107	0.0105	0.0177	0.0137	0.0002	0.0013	0.0022	0.0048	0.0228	0.0124
(35 infertile ;	median	0.0012 ^b^	0.0014 ^a^	0.0017 ^b^	0.0022 ^a^	0.0015 ^b^	0.0022 ^a^	0.0001	0.0002	0.0016 ^b^	0.0013 ^a^	0.0006 ^b^	0.0007 ^a^
20 fertile)	25th p	0.0004	0.0005	0.0009	0.0015	0.0005	0.0009	0.0001	0.0001	0.0004	0.0009	0.0002	0.0003
	75th p	0.0233	0.0156	0.0176	0.0089	0.0243	0.0157	0.0002	0.0003	0.0025	0.0024	0.0344	0.0137
**Rural**	mean	0.0004	0.0003	0.0011	0.0011	0.0009	0.0007	0.0002	0.0001	0.0009	0.0008	0.0003	0.0001
(22 infertile ;	median	0.0004 ^c^	0.0004 ^b^	0.0010 ^c^	0.0010 ^b^	0.0008 ^c^	0.0006 ^b^	0.0001	0.0001	0.0008 ^b^	0.0006 ^b^	0.0002 ^c^	0.0001 ^b^
8 fertile)	25th p	0.0003	0.0002	0.0006	0.0009	0.0005	0.0004	0.0001	0.0001	0.0004	0.0004	0.0001	0.0001
	75th p	0.0006	0.0004	0.0015	0.0012	0.0011	0.0008	0.0002	0.0002	0.0011	0.0014	0.0002	0.0002

* Indicates statistically significant different values between infertile and fertile women in the same area of residence (Mann-Whitney test corrected with the Bonferroni procedure). ^a,b,c^ Different superscript letters indicate statistically significant different values between areas within women of the same group (Mann-Whitney test corrected with the Bonferroni procedure). ^#^ 25th and 75th p indicate percentile values.

The mRNAs of the selected NRs were detected in all samples examined, therefore confirming the suitability of the NRs panel in PBMCs. Expression levels were comparable for ERα, ERβ, AR and PXR, while AhR and PPARγ were expressed at lower levels. 

Comparisons between infertile and fertile women within the areas showed that in the metropolitan area, the expression of ERα, ERβ, AR, AhR and PXR was approximately ten-fold (*p* < 0.01) higher in infertile women; on the contrary, in the other areas, no significant differences were found. 

The comparisons of NR expression levels between fertile women by area of residence showed that women from the metropolitan and urban areas had a comparable expression of ERα, ERβ, AR, AhR and PXR. Fertile women from the rural area had significantly lower expression of all these NRs compared to the urban area and of ERα and PXR compared to the metropolitan area. No difference was detected for PPARγ expression.

The comparisons of NRs expression levels between infertile women by area of residence showed a different picture. Infertile women from the metropolitan area displayed significantly higher expression levels of all NRs, but PPARγ, when compared to infertile women from the other two areas (*p* < 0.01 for both comparisons. Mean expression levels in the metropolitan area were about 10-(PXR) to 25-fold (AhR) higher than in the urban area and about 70-(AhR) to 900-fold (PXR) higher than in the rural area). 

## 4. Discussion

Our study, in the frame of the Italian project, PREVIENI, indicates the relationship between the residential area and biomarkers, serum/blood concentrations of some EDs and gene expression levels of a panel of NRs, in infertile and fertile women. In particular, BPA and MEHP levels, as well as expression of ERα, ERβ, AR, AhR and PXR were more prevalent in infertile women from the metropolitan area. In this area, BPA serum levels were also significantly higher in infertile women compared to fertile women. In the urban area, there was a noticeable increase of PFOA blood levels compared to the metropolitan area. This increase was detected also in the rural area, albeit to a lower extent; otherwise, women from the rural area overall showed the lowest values of MEHP and BPA serum concentrations, as well as of ERα, ERβ, AR, AhR and PXR expression. The presence of detectable PFOS blood levels was quite prevalent in the enrolled subjects, about 30%, but no significant relationship with the residential area was observed. Overall, the differences related to the residential area appeared more enhanced when comparing the infertile, rather than the fertile, groups.

The three areas represented quite distinct living environment scenarios according to selected territorial, demographic and productive indicators. The significantly higher levels of BPA (median levels and % >LOD) and MEHP (median levels) in women from the metropolitan area may reflect both the greater presence of economic activities employing these chemicals, as well as characteristic usage patterns of food commodities and consumer products. Moreover, in our study, BPA was the only ED specifically associated with infertility: the significant association between detectable BPA levels and infertility was confined to the metropolitan area, with an OR = 8.3. Furthermore, BPA levels in infertile women were about 30-fold higher than in controls of the same area.

In our study, we sampled BPA and MEHP in serum, so as to establish a more direct correlation with the biomarkers of effect measured in PBMCs. Indeed, we aimed at studying in the same matrix (*i.e*., blood) both biomarkers of exposure and NR expression identified as a potential, and toxicologically relevant, biomarker of effect for EDs [35]. Whereas blood is considered the matrix of choice for bioaccumulating compounds, like PFOS and PFOA, BPA and phthalates are regarded as EDs undergoing quick metabolism [36]. Nevertheless, several recent studies, using proper quality control analysis, have measured circulating levels of BPA and/or MEHP, as a toxicologically relevant DEHP metabolite, in humans, giving consistent evidence of their presence into the bloodstream. Studies using BPA and/or MEHP measurements in serum mainly address possible environment-health associations, such as the potential relationships with reproductive disorders [37,38], chronic diseases [39,40], breast cancer risk [41], as well as the risk estimation of BPA mother-fetus transfer [42].

In our study, we observed a widespread presence of BPA and MEHP in the bloodstream with significant differences related to the selected residence areas. The adopted protocols and devices for sampling and analysis allowed us to rule out any external contamination from plastics, lending further support to the meaningful correlation of the detected BPA and MEHP internal levels with the living environment in women with different reproductive health statuses [33,43].

We chose to determine total serum BPA, *i.e*., free together with glucuronated BPA, the major circulating metabolite [44]. We selected this approach taking into account the uncertainties on BPA metabolic fate in humans, recently discussed by Vandenberg *et al*. [45]. The consistent presence of detectable serum levels of BPA suggests a repeated and continuous uptake of the compound from aggregate exposure through both dietary and non-diet sources [25,46]. Concentrations detected in infertile women in the metropolitan area were higher compared to serum levels found in other HBM studies analyzing total BPA, mainly by ELISA methods [47], while those from the urban area were comparable to the reported ranges. A higher BPA exposure in the metropolitan area may also be consistent with the reported BPA increase in outdoor air in relation with the extent of urbanization [48].

Our findings are in agreement with studies demonstrating an association between BPA exposure and conditions associated with female infertility, such as polycystic ovary syndrome [28,49] and implantation failure in women undergoing IVF [26,50]. The available reports on endometriosis showed no significant correlation with urinary BPA [51], while a significant association with serum BPA is described [27,52]. Overall, our study points to BPA as a pollutant of concern in metropolitan scenarios, in terms of both exposure and reproductive health.

Results on MEHP indicate a generalized and continuous human exposure to DEHP, rapidly metabolized to MEHP [53]. Noteworthy, MEHP exposure was definitely higher (by an approximate 10-fold factor) in the metropolitan scenario compared to the other areas, both in fertile and infertile women. In our study, such generalized exposure was not associated with an increased risk of being infertile, also in the metropolitan area; however, we cannot conclude whether exposure levels were too low to show a detectable effect. Available data on MEHP serum levels in women of fertile age are limited: the values detected in our study were higher than in a Swedish population of women at delivery [54] and lower than in an Italian group of endometriotic women [20]. Epidemiological evidence on DEHP/MEHP reproductive effects in women is not univocal [20,21,22], differently from *in vivo* studies demonstrating MEHP’s adverse effects on fertility [23,55,56].

Women from the urban area, characterized also by a large presence of farm factories, had higher PFOA internal levels. Subjects from the rural area showed generally lower median concentrations for all of the EDs analyzed, which was expected from the characteristics of the territory; interestingly, however, 50% of women showed detectable PFOA levels, comparable to the urban areas. Eschauzier *et al*. [57] point out that perfluorinated alkylated acids, such as PFOA, are a contaminant of groundwater and water surface from different sources, hinting to a possible relationship with the water sources used in local agricultural activities. In fact, higher PFOA levels in the selected urban area of Ferrara may reflect a site-specific problem: PFOA pollution has been reported in the major water basin nearby the Ferrara area, the Po River, probably deriving from industrial sources [58]. Exposure to PFOS showed no difference among the areas, as well between infertile and fertile women. Unlike PFOA, PFOS exposure is almost completely related to food chain contamination [7,10]. Therefore, our findings may indicate a comparable PFOS dietary exposure in the selected population. PFOS and PFOA concentrations observed in our study were, on average, lower than estimated reference values for the German population [59], but comparable to levels found in Catalonia [60] and Italy [61].

Similarly to biomarkers of ED exposure, the area-stratified analysis showed significant differences as regards the gene expression levels of the NR panel, except for PPARγ, which did not differ either in relation to the health status or to the area of residence. Interestingly, women from the rural area generally presented significantly lower gene expression levels, with some difference between fertile and infertile women: although no conclusion can be made based on the present data, it is noteworthy that this finding seems to parallel the overall lower EDs exposure level in the same area. 

In infertile women, the NR gene expression significantly differed among areas, with the highest levels in women from the metropolitan area followed by those from urban and rural area. Indeed, infertile women from metropolitan area presented a significant 10-fold increase, with respect to fertile women, in the gene expression levels of five out of six analyzed NRs, namely ERα, ERβ, AR, AhR and PXR. These NRs were all positively correlated, indicating a common responsiveness. 

In our previous study, we showed a positive correlation between such NRs and BPA internal levels [31]. Since BPA serum levels were markedly higher in infertile women from the metropolitan area, it is plausible that this ED could contribute to the observed NRs increase in this group. Furthermore, MEHP showed a positive correlation with the same NR panel [31]. Even though MEHP internal levels showed no significant relationship with the fertility status, the internal levels in infertile women from the metropolitan area were significantly higher compared to the other areas. Therefore, it is plausible that MEHP might have added up to the BPA effect on NR expression in the study group of infertile women of the metropolitan area. The possible impact, if any, of an MEHP-related effect on NR expression in women remains to be ascertained. 

We recognize that our data do not provide direct evidence of a causal link between ED serum levels and NR expression in PBMCs, including a possible combined effect of the simultaneous exposure to the EDs investigated. The metropolitan living environment is both associated with higher serum levels of BPA and MEHP and with an inducing effect on ERs and AR expression in infertile women. BPA is considered an ER-agonist, as well as an AR antagonist [29]; MEHP may display anti-estrogenic and anti-androgenic activities acting on steroid synthesis rather than on ERs or AR [62]. Indeed, NR expression may be modulated by direct, as well as cross-talk or feed-back mechanisms [63]. The observed increase of AhR and PXR is consistent with the available evidence on both BPA and MEHP mechanisms [23,29,30,64,65]. However, due to the lack of information on the expression of NRs in PBMCs to compare with, we may only hypothesize that the increase may become evident only when exposure to expression-inducer compound(s) exceeds a certain threshold.

It is noteworthy that the more persistent substances, PFOS and PFOA, showed no association with fertility status in our study, contrary to the findings of some previous studies [11,12,13]. As previously noted, based on the limited data available, PFOS and PFOA internal levels were comparable to those found in the general population of Mediterranean Europe [60,61] and lower than reference values for the Germany population [59]. Therefore, our negative findings may simply reflect the lack of effect by a baseline exposure level.

While acknowledging the unavoidable limitations of cross-sectional studies, our results clearly point out the characteristics of the area of residence as a relevant factor when investigating environmental exposures, such as EDs. In the present study, the metropolitan area emerged as a hotspot for BPA and MEHP exposure, BPA being significantly associated with an increased risk of being infertile in women. Moreover, our results point out a panel of NRs (ERα, ERβ, AR, AhR and PXR) induced at the same time in PBMCs of infertile subjects. PBMCs are responsive to estrogens, natural ligand of ERs, which mediate the immune cells response and PBMC infiltration in tissues. Indeed, cardiovascular disorders and autoimmune diseases are associated with the presence of PBMCs in tissues. Notably, in reproductive female organs, PBMCs regulate the extracellular matrix and are implicated in several functions, such as ovulation, menstruation and implantation [66]. Thus, investigations in reproductive target tissues may be warranted. The identified panel of NRs can be further developed as a suitable biomarker of effect when evaluating ED exposure in relation to reproductive health.

## 5. Conclusions

Our study reinforces the concept that humans are continuously exposed to several EDs, still widely present in consumer goods, and that this may represent a risk factor for woman’s fertility in relation to areas and living environment scenarios. Further research is warranted on the potential interactions of the internal burdens of EDs acting on similar pathways/targets, also expanding the range of EDs under scrutiny.

## References

[1] Zegers-Hochschild F., Adamson G.D., de Mouzon J., Ishihara O., Mansour R., Nygren K., Sullivan E., van der Poel S., International Committee for Monitoring Assisted Reproductive Technology, World Health Organization (2009). The International Committee for Monitoring Assisted Reproductive Technology (ICMART) and the World Health Organization (WHO) revised glossary on ART terminology, 2009. Hum. Reprod..

[2] Mascarenhas M.N., Flaxman S.R., Boerma T., Vanderpoel S., Stevens G.A. (2012). National, regional, and global trends in infertility prevalence since 1990: A systematic analysis of 277 health surveys. PLoS Med..

[3] Caserta D., Maranghi L., Mantovani A., Marci R., Maranghi F., Moscarini M. (2008). Impact of endocrine disruptor chemicals in gynaecology. Hum. Reprod. Update.

[4] Caserta D., Mantovani A., Marci R., Fazi A., Ciardo F., La Rocca C., Maranghi F., Moscarini M. (2011). Environment and women’s reproductive health. Hum. Reprod. Update.

[5] Bonde J.P., Toft G., Rylander L., Rignell-Hydbom A., Giwercman A., Spano M., Manicardi G.C., Bizzaro D., Ludwicki J.K., Zvyezday V. (2008). Fertility and markers of male reproductive function in inuit and European populations spanning large contrasts in blood levels of persistent organochlorines. Environ. Health Perspect..

[6] Wojtyniak B.J., Rabczenko D., Jonsson B.A., Zvezday V., Pedersen H.S., Rylander L., Toft G., Ludwicki J.K., Goralczyk K., Lesovaya A. (2010). Association of maternal serum concentrations of 2,2’, 4,4’5,5’-Hexachlorobiphenyl (CB-153) and 1,1-Dichloro-2,2-Bis (P-Chlorophenyl)-Ethylene (P,P’-DDE) levels with birth weight, gestational age and preterm births in inuit and European populations. Environ. Health.

[7] Benford D., de Boer J., Carere A., di Domenico A., Johansson N., Schrenk D., Schoeters G., de Voogt P., Dellatte E. (2008). Opinion of the scientific panel on contaminants in the food chain on perfluorooctane sulfonate (PFOS), perfluorooctanoic acid (PFOA) and their salts. EFSA J..

[8] United Nations Environment Programme (2010). Stockholm Convention on Persistent Organic Pollutants. The 9 New POPs; COP 4.

[9] European Food Safety Authority (2011). Results of the monitoring of perfluoroalkylated substances in food in the period 2000–2009. EFSA J..

[10] European Food Safety Authority (2012). Perfluoroalkylated substances in food: Occurrence and dietary exposure. EFSA J..

[11] Governini L., Orvieto R., Guerranti C., Gambera L., de Leo V., Piomboni P. (2011). The impact of environmental exposure to perfluorinated compounds on oocyte fertilization capacity. J. Assist. Reprod. Genet..

[12] Fei C., McLaughlin J.K., Lipworth L., Olsen J. (2009). Maternal levels of perfluorinated chemicals and subfecundity. Hum. Reprod..

[13] Louis G.M., Peterson C.M., Chen Z., Hediger M.L., Croughan M.S., Sundaram R., Stanford J.B., Fujimoto V.Y., Varner M.W., Giudice L.C. (2012). Perfluorochemicals and endometriosis: The ENDO study. Epidemiology.

[14] Benninghoff A.D., Bisson W.H., Koch D.C., Ehresman D.J., Kolluri S.K., Williams D.E. (2011). Estrogen-Like activity of perfluoroalkyl acids *in vivo* and interaction with human and rainbow trout estrogen receptors *in vitro*. Toxicol. Sci..

[15] Bjork J.A., Wallace K.B. (2009). Structure-activity relationships and human relevance for perfluoroalkyl acid-induced transcriptional activation of peroxisome proliferation in liver cell cultures. Toxicol. Sci..

[16] Takacs M.L., Abbott B.D. (2007). Activation of mouse and human peroxisome proliferator-activated receptors (alpha, beta/delta, gamma) by perfluorooctanoic acid and perfluorooctane sulfonate. Toxicol. Sci..

[17] Ren H., Vallanat B., Nelson D.M., Yeung L.W.Y., Guruge K.S., Lam P.K.S., Lehman-McKeeman L.D., Corton J.C. (2009). Evidence for the involvement of xenobiotic-responsive nuclear receptors in transcriptional effects upon perfluoroalkyl acid exposure in diverse species. Reprod. Toxicol..

[18] Latini G. (2005). Monitoring phthalate exposure in humans. Clin. Chim. Acta.

[19] European Commission (2011). Commission Regulation (EU) No. 10/2011 of 14 January 2011 on plastic materials and articles intended to come into contact with food. Off. J. Eur. Union L.

[20] Cobellis L., Latini G., de Felice C., Razzi S., Paris I., Ruggieri F., Mazzeo P., Petraglia F. (2003). High plasma concentrations of di-(2-ethylhexyl)-phthalate in women with endometriosis. Hum. Reprod..

[21] Itoh H., Iwasaki M., Hanaoka T., Sasaki H., Tanaka T., Tsugane S. (2009). Urinary phthalate monoesters and endometriosis in infertile Japanese women. Sci. Total Environ..

[22] Kim S.H., Chun S., Jang J.Y., Chae H.D., Kim C., Kang B.M. (2011). Increased plasma levels of phthalate esters in women with advanced-stage endometriosis: A prospective case-control study. Fertil. Steril..

[23] Lovekamp-Swan T., Davis B.J. (2003). Mechanisms of phthalate ester toxicity in the female reproductive system. Environ. Health Perspect..

[24] Ritter S. (2011). Debating BPA’s toxicity. Chem. Eng. News.

[25] European Food Safety Authority (2014). Draft scientific opinion on the risks to public health related to the presence of bisphenol A (BPA) in foodstuffs. EFSA J..

[26] Ehrlich S., Williams P.L., Missmer S.A., Flaws J.A., Berry K.F., Calafat A.M., Ye X., Petrozza J.C., Wright D., Hauser R. (2012). Urinary bisphenol a concentrations and implantation failure among women undergoing *in vitro* fertilization. Environ. Health Perspect..

[27] Cobellis L., Colacurci N., Trabucco E., Carpentiero C., Grumetto L. (2009). Measurement of bisphenol A and bisphenol B levels in human blood sera from healthy and endometriotic women. Biomed. Chromatogr..

[28] Kandaraki E., Chatzigeorgiou A., Livadas S., Palioura E., Economou F., Koutsilieris M., Palimeri S., Panidis D., Diamanti-Kandarakis E. (2011). Endocrine disruptors and polycystic ovary syndrome (PCOS): Elevated serum levels of bisphenol A in women with PCOS. J. Clin. Endocrinol. Metab..

[29] Rubin B.S. (2011). Bisphenol A: An endocrine disruptor with widespread exposure and multiple effects. J. Steroid Biochem. Mol. Biol..

[30] Sui Y., Ai N., Park S.H., Rios-Pilier J., Perkins J.T., Welsh W.J., Zhou C. (2012). Bisphenol A and its analogues activate human pregnane X receptor. Environ. Health Perspect..

[31] Caserta D., Ciardo F., Bordi G., Guerranti C., Fanello E., Perra G., Borghini F., La Rocca C., Tait S., Bergamasco B. (2013). Correlation of endocrine disrupting chemicals serum levels and white blood cells gene expression of nuclear receptors in a population of infertile women. Int. J. Endocrinol..

[32] Governini L., Guerranti C., Focardi S., Cuppone A., Stendardi A., de Leo V., Piomboni P. (2009). Impact of environmental exposure to perfluorinated compounds on sperm DNA quality. Syst. Biol. Reprod. Med..

[33] Vandenberg L.N., Gerona R.R., Kannan K., Taylor J.A., van Breemen R.B., Dickenson C.A., Liao C., Yuan Y., Newbold R.R., Padmanabhan V. (2014). A round robin approach to the analysis of bisphenol a (BPA) in human blood samples. Environ. Health.

[34] European Food Safety Authority (2010). Management of left-censored data in dietary exposure assessment of chemical substances. EFSA J..

[35] Wens B., de Boever P., Verbeke M., Hollanders K., Schoeters G. (2013). Cultured human peripheral blood mononuclear cells alter their gene expression when challenged with endocrine-disrupting chemicals. Toxicology.

[36] Koch H.M., Calafat A.M. (2009). Human body burdens of chemicals used in plastic manufacture. Philos. Trans. R. Soc. Lond. B Biol. Sci..

[37] Lathi R.B., Liebert C.A., Brookfield K.F., Taylor J.A., vom Saal F.S., Fujimoto V.Y., Baker V.L. (2014). Conjugated bisphenol A (BPA) in maternal serum in relation to miscarriage risk. Fertil. Steril..

[38] Specht I.O., Toft G., Hougaard K.S., Lindh C.H., Lenters V., Jönsson B.A., Heederik D., Giwercman A., Bonde J.P.E. (2014). Associations between serum phthalates and biomarkers of reproductive function in 589 adult men. Environ. Int..

[39] Lind P.M., Zethelius B., Lind L. (2012). Circulating levels of phthalate metabolites are associated with prevalent diabetes in the elderly. Diabetes Care.

[40] Olsén L., Lind L., Lind P.M. (2012). Associations between circulating levels of bisphenol A and phthalate metabolites and coronary risk in the elderly. Ecotoxicol. Environ. Saf..

[41] Sprague B.L., Trentham-Dietz A., Hedman C.J., Wang J., Hemming J.D., Hampton J.M., Buist D.S., Bowles E.J.A., Sisney G.S., Burnside E.S. (2013). Circulating serum xenoestrogens and mammographic breast density. Breast Cancer Res..

[42] Aris A. (2014). Estimation of bisphenol A (BPA) concentrations in pregnant women, fetuses and nonpregnant women in eastern townships of Canada. Reprod. Toxicol..

[43] Ye X., Zhou X., Hennings R., Kramer J., Calafat A.M. (2013). Potential external contamination with bisphenol A and other ubiquitous organic environmental chemicals during biomonitoring analysis: An elusive laboratory challenge. Environ. Health Perspect..

[44] Völkel W., Colnot T., Csanády G.A., Filser J.G., Dekant W. (2002). Metabolism and kinetics of bisphenol A in humans at low doses following oral administration. Chem. Res. Toxicol..

[45] Vandenberg L.N., Hunt P.A., Myers J.P., vom Saal F.S. (2013). Human exposures to bisphenol A: Mismatches between data and assumptions. Rev. Environ. Health.

[46] Stahlhut R.W., Welshons W.V., Swan S.H. (2009). Bisphenol A data in NHANES suggest longer than expected half-life, substantial nonfood exposure, or both. Environ. Health Perspect..

[47] Vandenberg L.N., Chahoud I., Heindel J.J., Padmanabhan V., Paumgartten F.J., Schoenfelder G. (2010). Urinary, circulating, and tissue biomonitoring studies indicate widespread exposure to bisphenol A. Environ. Health Perspect..

[48] Fu P., Kawamura K. (2010). Ubiquity of bisphenol A in the atmosphere. Environ. Pollut..

[49] Takeuchi T., Tsutsumi O., Ikezuki Y., Takai Y., Taketani Y. (2004). Positive relationship between androgen and the endocrine disruptor, bisphenol A, in normal women and women with ovarian dysfunction. Endocr. J..

[50] Ehrlich S., Williams P.L., Missmer S.A., Flaws J.A., Ye X., Calafat A.M., Petrozza J.C., Wright D., Hauser R. (2012). Urinary bisphenol a concentrations and early reproductive health outcomes among women undergoing IVF. Hum. Reprod..

[51] Itoh H., Iwasaki M., Hanaoka T., Sasaki H., Tanaka T., Tsugane S. (2007). Urinary bisphenol-A concentration in infertile Japanese women and its association with endometriosis: A cross-sectional study. Environ. Health Prev. Med..

[52] Caserta D., Bordi G., Ciardo F., Marci R., La Rocca C., Tait S., Bergamasco B., Stecca L., Mantovani A., Guerranti C. (2013). The influence of endocrine disruptors in a selected population of infertile women. Gynecol. Endocrinol..

[53] Koch H., Bolt H., Angerer J. (2004). Di (2-Ethylhexyl) phthalate (DEHP) metabolites in human urine and serum after a single oral dose of deuterium-labelled DEHP. Arch. Toxicol..

[54] Högberg J., Hanberg A., Berglund M., Skerfving S., Remberger M., Calafat A.M., Filipsson A.F., Jansson B., Johansson N., Appelgren M. (2008). Phthalate diesters and their metabolites in human breast milk, blood or serum, and urine as biomarkers of exposure in vulnerable populations. Environ. Health Perspect..

[55] Reinsberg J., Wegener-Toper P., van der Ven K., van der Ven H., Klingmueller D. (2009). Effect of mono-(2-ethylhexyl) phthalate on steroid production of human granulosa cells. Toxicol. Appl. Pharmacol..

[56] Gupta R.K., Singh J.M., Leslie T.C., Meachum S., Flaws J.A., Yao H.H. (2010). Di-(2-Ethylhexyl) phthalate and mono-(2-ethylhexyl) phthalate inhibit growth and reduce estradiol levels of antral follicles *in vitro*. Toxicol. Appl. Pharmacol..

[57] Eschauzier C., Raat K.J., Stuyfzand P.J., de Voogt P. (2013). Perfluorinated alkylated acids in groundwater and drinking water: Identification, origin and mobility. Sci. Total Environ..

[58] Loos R., Locoro G., Huber T., Wollgast J., Christoph E.H., de Jager A., Manfred Gawlik B., Hanke G., Umlauf G., Zaldívar J. (2008). Analysis of perfluorooctanoate (PFOA) and other perfluorinated compounds (PFCs) in the River Po Watershed in N-Italy. Chemosphere.

[59] Wilhelm M., Angerer J.R., Fromme H., Hölzer J. (2009). Contribution to the evaluation of reference values for PFOA and PFOS in plasma of children and adults from Germany. Int. J. Hyg. Environ. Health.

[60] Ericson I., Gómez M., Nadal M., van Bavel B., Lindström G., Domingo J.L. (2007). Perfluorinated chemicals in blood of residents in Catalonia (Spain) in relation to age and gender: A pilot study. Environ. Int..

[61] Ingelido A.M., Marra V., Abballe A., Valentini S., Iacovella N., Barbieri P., Porpora M.G., Domenico A.d., Felip E.D. (2010). Perfluorooctanesulfonate and perfluorooctanoic acid exposures of the Italian general population. Chemosphere.

[62] Lee H.J., Chattopadhyay S., Gong E.Y., Ahn R.S., Lee K. (2003). Antiandrogenic effects of bisphenol A and nonylphenol on the function of androgen receptor. Toxicol. Sci..

[63] Guo C., Ni X., Zhu P., Li W., Zhu X., Sun K. (2010). Induction of progesterone receptor A form attenuates the induction of cytosolic phospholipase a2alpha expression by cortisol in human amnion fibroblasts. Reproduction.

[64] Hurst C.H., Waxman D.J. (2004). Environmental phthalate monoesters activate pregnane X receptor-mediated transcription. Toxicol. Appl. Pharmacol..

[65] Mnif W., Pascussi J.M., Pillon A., Escande A., Bartegi A., Nicolas J.C., Cavailles V., Duchesne M.J., Balaguer P. (2007). Estrogens and antiestrogens activate hPXR. Toxicol. Lett..

[66] Stygar D., Westlund P., Eriksson H., Sahlin L. (2006). Identification of wild type and variants of oestrogen receptors in polymorphonuclear and mononuclear leucocytes. Clin. Endocrinol. Oxf..

